# Occurrence and identification of risk areas of *Ixodes ricinus*-borne pathogens: a cost-effectiveness analysis in north-eastern Italy

**DOI:** 10.1186/1756-3305-5-61

**Published:** 2012-03-27

**Authors:** Gioia Capelli, Silvia Ravagnan, Fabrizio Montarsi, Silvia Ciocchetta, Stefania Cazzin, Elena Porcellato, Amira Mustafa Babiker, Rudi Cassini, Annalisa Salviato, Giovanni Cattoli, Domenico Otranto

**Affiliations:** 1 Istituto Zooprofilattico Sperimentale delle Venezie, Viale dell'Università, 10, 35020, Legnaro (Pd), Italy; 2 Dipartimento di Scienze Sperimentali Veterinarie, Università degli Studi di Padova, 35020 Legnaro, Padova, Italy; 3 Dipartimento di Sanità Pubblica e Zootecnia, Università degli Studi di Bari, 70010 Valenzano, Bari, Italy

**Keywords:** *Ixodes ricinus*, tick-borne diseases, surveillance, economic evaluation, Italy.

## Abstract

**Background:**

*Ixodes ricinus*, a competent vector of several pathogens, is the tick species most frequently reported to bite humans in Europe. The majority of human cases of Lyme borreliosis (LB) and tick-borne encephalitis (TBE) occur in the north-eastern region of Italy. The aims of this study were to detect the occurrence of endemic and emergent pathogens in north-eastern Italy using adult tick screening, and to identify areas at risk of pathogen transmission. Based on our results, different strategies for tick collection and pathogen screening and their relative costs were evaluated and discussed.

**Methods:**

From 2006 to 2008 adult ticks were collected in 31 sites and molecularly screened for the detection of pathogens previously reported in the same area (i.e., LB agents, TBE virus, *Anaplasma phagocytophilum, Rickettsia *spp., *Babesia *spp., "*Candidatus Neoehrlichia mikurensis*"). Based on the results of this survey, three sampling strategies were evaluated *a*-*posteriori*, and the impact of each strategy on the final results and the overall cost reductions were analyzed. The strategies were as follows: tick collection throughout the year and testing of female ticks only (strategy A); collection from April to June and testing of all adult ticks (strategy B); collection from April to June and testing of female ticks only (strategy C).

**Results:**

Eleven pathogens were detected in 77 out of 193 ticks collected in 14 sites. The most common microorganisms detected were *Borrelia burgdorferi *sensu lato (17.6%), *Rickettsia helvetica *(13.1%), and "*Ca. N. mikurensis*" (10.5%). Within the *B. burgdorferi *complex, four genotypes (i.e., *B. valaisiana, B. garinii, B. afzelii*, and *B. burgdorferi *sensu stricto) were found. Less prevalent pathogens included *R. monacensis *(3.7%), TBE virus (2.1%), *A. phagocytophilum *(1.5%), *Bartonella *spp. (1%), and *Babesia *EU1 (0.5%). Co-infections by more than one pathogen were diagnosed in 22% of infected ticks. The prevalences of infection assessed using the three alternative strategies were in accordance with the initial results, with 13, 11, and 10 out of 14 sites showing occurrence of at least one pathogen, respectively. The strategies A, B, and C proposed herein would allow to reduce the original costs of sampling and laboratory analyses by one third, half, and two thirds, respectively. Strategy B was demonstrated to represent the most cost-effective choice, offering a substantial reduction of costs, as well as reliable results.

**Conclusions:**

Monitoring of tick-borne diseases is expensive, particularly in areas where several zoonotic pathogens co-occur. Cost-effectiveness studies can support the choice of the best monitoring strategy, which should take into account the ecology of the area under investigation, as well as the available budget.

## Background

Ticks are second only to mosquitoes as vectors of zoonotic pathogens and are recognized as the primary vectors of vector-borne diseases in temperate climates [1].

*Ixodes ricinus *(Acari: Ixodidae), also known as "wood", "sheep" or "castor-bean" tick, is the ixodid species most frequently reported to bite humans in Europe [2], and acts as a major vector of viral, bacterial, and protozoan agents, which infect many domesticated and wild animals, as well as humans [3]. For instance, this species can transmit the tick-borne encephalitis virus (TBEv), *Borrelia burgdorferi *sensu lato (s.l.), the aetiological agent of Lyme borreliosis (LB), as well as other pathogens, e.g. *Rickettsia, Anaplasma *and *Babesia *spp. [4]. The distribution of tick-transmitted pathogens (TTPs) is primarily dependent on tick density and the availability of animal reservoirs. *I. ricinus *acts as vector of several pathogens mostly because of its large host *spectrum*, being able to feed on more than 300 animal species [2].

In Italy, *I. ricinus *occurs throughout the peninsula and its populations reach the highest density in hilly and pre-alpine northern areas, characterized by a temperate climate, with cold winters, and cool and humid summers [5]. These areas represent the optimal *I. ricinus *biotope, consisting of microhabitats characterized by humidity above 85% and a well conserved biocenosis of wild animals (including small and large mammals, birds, and reptiles). The north-eastern region of Italy accounts for the majority of human cases of LB and TBE [6]; the first cases of Human Granulocytic Anaplasmosis (HGA) by *Anaplasma phagocytophilum *have also been reported in the same area [7,8].

According to Heiman *et al. *[1], tick-borne diseases (TBDs) are also likely to become among the infectious threats, one of the main concerns for public health in Europe within the coming years; therefore, well planned, efficient, and cost-effective surveillance systems need to be implemented. The first step towards planning TBDs surveillance should consist in assessing the panel of pathogens occurring in a given area and their relative epidemiological importance, in relation to their prevalence in vectors and hosts and the severity of the diseases that they cause. Alongside burden of pathogens, information on vector density and dynamics also needs to be aquired. In order to assess the spatial and temporal distribution of *I. ricinus *and the environmental factors associated with its occurrence in north-eastern Italy, the Ministry of Health launched a three year-project (code RC-IZSVe 11/04), whose results have been published elsewhere [9,10]. In the present study, adult ticks collected through the previous years were screened for all the pathogens known or suspected to occur in north-eastern Italy, including TBEv, LB agents, *A. phagocytophilum, Rickettsia *spp., *Babesia *spp. and the recently described bacterium "*Candidatus Neoehrlichia mikurensis*".

The aims of this study were to assess the suitability of adult tick screening for (i) detecting the occurrence of endemic and emergent pathogens in north-eastern Italy, and (ii) identifying areas at risk for pathogen transmission to animals and humans. Based on the results of this survey, different strategies for collection of ticks and pathogen screening, as well as their relative costs, were evaluated and discussed.

Over the past few years, central and local Governments have drastically reduced funds to the majority of institutions involved in monitoring vector-borne diseases. This will inevitably impact on ways of approaching research and surveillance actions in terms of sampling design, and data collection and analyses.

## Methods

### Study area

**Figure 1:**
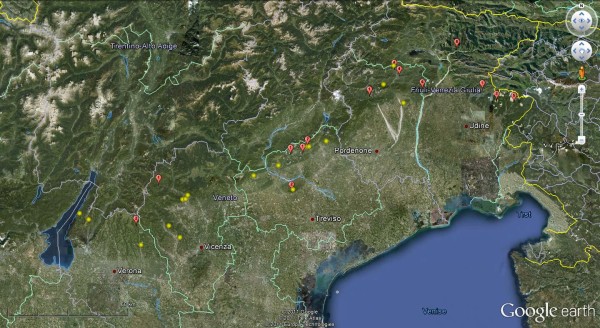
**Map of north-eastern Italy showing the 31 sites in which adult ticks were found (yellow: sites negative for pathogens; red: sites positive for one or more pathogens; number of pathogens/site is also reported within each red symbol)**.

From 2006 to 2008, *I. ricinus *ticks were collected in an area of north-eastern Italy (45°30'52"N to 46°32'4"N and 11°9'52"E to 13°1'14"E) within the regions of Veneto and Friuli Venezia Giulia (FVG), including five provinces (i.e., Vicenza, Verona, Treviso, Pordenone, and Udine) (Figure 1). Sampling was carried out in the south-eastern slope of hilly and pre-alpine areas in habitats suitable for growth and survival of *I. ricinus*, characterized by heterogeneous deciduous woodland and mixed forest, and occurrence of domestic and/or wild animals. The altitudes of the sites investigated ranged between 120 and 1308 m above sea level (a.s.l.). All sites were close to human dwellings or easily accessible through footpaths.

### Tick sampling and identification

From 2006 to 2008 a permanent site for each province was monitored monthly, whereas another 50 sites were monitored on one occasion each month (herein after defined as temporary sites). Ticks were collected by dragging using a 1 m^2 ^white flannel cloth, through 50 m transects, stopping every 2.5 m to prevent their detachment. Once collected, ticks were kept refrigerated at + 4°C, counted, grouped according to their developmental stage, and identified based on morphological features [11]. All adults collected throughout the three years at 31 sites (5 permanents and 26 temporary) were molecularly processed.

### Biomolecular analyses for the identification of pathogens and sequencing

Nucleic acids were extracted from single adult ticks using All Prep DNA/RNA mini Kit (Qiagen, Inc., Valencia, CA), according to the manufacturer's instructions and then kept frozen at -80°C. Target genes, primers, and probes used for testing and the size of the PCR amplification products are listed in Table 1 and 2.

**Table 1:** Biomolecular method used for pathogen identification, target genes, primers, probes and references.

Species	method	gene	primers	Nucleotide sequence (5'- 3')	Amplicon **size (bp)**^**c**^	**Ref**.
Ixodes	PCR	16S ribosomal RNA	F-16sIxodes	AAAAAAATACTCTAGGGATAACAGCGTAA	97	[12]
(extraction control)			R-16sIxodes	ACCAAAAAAGAATCCTAATCCAACA		
			16s-Ixodes-Probe	TTTTGGATAGTTCATATAGATAAAATAGTTTGC GACCTCG		
*B. burgdorferi s.l*.	real time PCR (duplex)	23S-rRNA	Bb23Sf	CGAGTCTTAAAAGGGCGATTTAGT	75	[14]
			Bb23Sr	GCTTCAGCCTGGCCATAAATAG		
			Bb23Sp-FAM	AGATGTGGTAGACCCGAAGCCGAGTG		
*A. phagocytophilum*	real time PCR (duplex)	msp2	ApMSP2f	ATGGAAGGTAGTGTTGGTTATGGTATT	77	[14]
			ApMSP2r	TTGGTCTTGAAGCGCTCGTA		
			ApMSP2p-HEX	TGGTGCCAGGGTTGAGCTTGAGATTG		
*B. burgdorferi s.l*.	PCR	flagellin	FLA1	AGAGCAACTTACAGACGAAATTAAT	482	[16]
			FLA2	CAAGTCTATTTTGGAAAGCACCTAA		
*A. phagocytophilum*	PCR	msp2	msp2-3f	CCAGCGTTTAGCAAGATAAGAG	334	[15]
			msp2-3r	GMCCAGTAACAACATCATAAGC		
TBEv	rRT-PCR	3' non-coding region	F-TBE 1	GGGCGGTTCTTGTTCTCC	67	[12]
			R-TBE 1	ACACATCACCTCCTTGTCAGACT		
			TBE-Probe-WT	TGAGCCACCATCACCCAGACACA		
TBEv	nested PCR	non-structural protein NS5	FSM-1	GAGGCTGAACAACTGCACGA	357	[13]
			FSM-2	GAACACGTCCATTCCTGATCT		
		non-structural protein NS5	FSM-1i	ACGGAACGTGACAAGGCTAG	251	
			FSM-2i	GCTTGTTACCATCTTTGGAG		
*Rickettsia spp*.	PCR	citrate synthase	RpCS.877p	GGGGGCCTGCTCACGGCGG	381	[17]
			RpCS1258n	ATTGCAAAAAGTACAGTGAACA		
*Cand. N. mikurensis*	PCR	groEL	NM-128s	AACAGGTGAAACACTAGATAAGTCCAT	1024	[19]
			NM-1152as	TTCTACTTTGAACATTTGAAGAATTACTAT		
Babesia/Theileria	PCR	18S rRNA	RLB-F2	GACACAGGGAGGTAGTGACAA	400	[18]
			RLB-R2	CTAAGAATTTCACCTCTGACAGT		

**Table 2:** Primers and UPL used for genospecies identification of *Borrelia burgdorferi *s.l. in co-infected ticks using real time PCR assays

*Genospecies*	*Target gene*	*5'→ 3'primer sequence*	*UPL number*	Amplicon size (bp)*^c^*
*B. burgdorferi *s.s.	OspA^a^	TCTTGAAGGAACTTTAACTGCTGA TGAAACTTCCCCAGATTTTGA	#119	***97***
*B. afzelii*	OspA	GACTCCGCAGGTACCAATTT AAAGCGTTTTTAAGTTCATCAAGTG	#98	***71***
*B. garinii*	Fla^b^	TCTGCTATGATTATGCCACCA CCTTTGCCTAAGAATTGATTACCA	#2	***74***
*B. valaisiana*	Fla	CCAAATGCACATGTTGTCAAA TTTGCAGGTTGCATTCCA	#132	**78**

^a^OspA: Outer surface protein A gene; ^b^Fla: flagellin gene; ^c^bp: base pairs

To ensure the effectiveness of the nucleic acid extraction, a real time PCR targeting the 16S rRNA was applied [12].

A real time PCR was used for TBEV detection [12]. Positive results in real-time PCR were confirmed by a nested real time (RT)-PCR [13]. A multiplex RT-PCR was used for the simultaneous detection of *A. phagocytophilum *and *B. burgdorferi *s.l. [14].

All samples positive for *A. phagocytophilum *were confirmed by a specific PCR [15] and sequenced. To determine the genospecies of *B. burgdorferi *s.l., a conserved region of the flagellin gene was amplified by PCR for all the *B. burgdorferi *s.l. positive samples according to a protocol previously published [16], followed by genetic sequencing of the PCR products. Sequence electropherograms of *B. burgdorferi *s.l. were checked for quality and to reveal the presence of double nucleotide peaks. When double peaks were detected in both (i.e., for primers forward and reverse) high-quality sequence electropherograms and their location corresponded to the variable sites specific for a certain genospecies, a multiple infection was suspected. To confirm the presence of co-infections of *B. burgdorferi *genospecies, four RT-PCR assays were performed by using Universal Probe Library (UPL) (Roche, Mannheim, Germany), presynthesized, fluorescence-labelled locked nucleic acid (LNA) hydrolysis probes, to detect specifically *B. burgdorferi *s.s., *B. afzelii, B. garinii *and *B. valaisiana*. Primers and probes number (Table 2) were chosen by free online software (UPL Assay Design Center web service; https://www.roche-applied-science.com) and the UPL probe from the Roche Universal Probe Library collection. Real time PCR was performed with a reaction mixture consisting of 2 μl of DNA, 5 μl of 2× Light Cycler 480 Probes Master (Roche, Mannheim, Germany), 300 nM of each *Borrelia *species primer set and 200 nM of each corresponding UPL probe with a thermal cycling profile consisting of an initial activation at 95°C for 10 min, followed by 45 cycles of denaturation at 95°C for 10 s and annealing/extension at 60°C for 30 s and a final cooling step at 40°C for 30 s. Fluorescence data were collected in the annealing/extension phase at 60°C.

*Rickettsia *spp., *Babesia *spp., and "*Ca. N. mikurensis" *were amplified with protocols described in the literature [17-19] and the species identity determined by genetic sequencing.

RT-PCRs were carried out on a Rotor Gene 6000 real-Time PCR system (Corbett, Australia) and traditional PCRs on a GeneAmp^®^PCR System 9700 thermal cycler (Applied Biosystems, Foster City, CA).

All PCR products were sequenced using the Big Dye Terminator v 3.1 cycle sequencing kit (Applied Biosystem, Foster City, CA, USA). The products of the sequencing reactions were purified using PERFORMA DTR Ultra 96-Well kit (Edge BioSystems, Gaithersburg, MD, USA) and sequenced in a 16-capillary ABI PRISM 3130 × l Genetic Analyzer (Applied Biosystem, Foster City, CA, USA). Sequence data were assembled and edited with SeqScape software v 2.5 (Applied Biosystem, Foster City, CA, USA), aligned and compared with representative sequences available in GenBank.

### Statistical analysis

Differences in the prevalence of pathogens in relation to tick gender, province/region of origin, and month/year of collection were tested by using χ^2 ^or Fisher's exact test, when appropriate. The correlation between number of adults examined and number of pathogens recovered was tested by linear regression. The software used was SPSS (SPSS Inc., Chicago, IL) for windows, version 13.0.

### Cost estimation

Costs of molecular procedures were calculated as described by Cattoli *et al. *[20], adjusted for DNA/RNA extraction used in this study. Travel costs (distance range from the sites, i.e., 62-218 km), included fuel, tolls and meals for staff involved in tick collections. Costs for staff were calculated based on the number of working days and on staff salary scales of Istituto Zooprofilattico Sperimentale delle Venezie, Italy (2011).

## Results

### Occurrence of pathogens and co-infections

During the 146 dragging collections performed throughout the three years, 193 adult ticks (i.e., 95 females and 97 males) were collected in 31 sites (range 1-47 ticks per site). At least one pathogen was detected in 77 (39.9%) ticks from 14 sites (45%). Overall, 11 pathogens were identified with variable prevalence (Table 3), with *B. burgdorferi *s.l. the most common (17.6%), followed by *R. helvetica *(13.1%) and "*Ca. N. mikurensis" *(10.5%). Four genotypes within the *B. burgdorferi *complex (i.e., *B. valaisiana, B. garinii, B. afzelii*, and *B. burgdorferi *sensu stricto) were identified. TBE virus, *A. phagocytophilum, R. monacensis*, and *Babesia *EU1 (proposed name *B. venatorum*) were detected more rarely (Table 3). GenBank accession numbers of the most representative sequences are reported in Table 3.

**Table 3:** Pathogens and their prevalence (P) detected in 193 adult *Ixodes ricinus *from 2006 to 2008 in north-eastern Italy, permanent and temporary sites positives and year of detection.

Pathogens [accession numbers]	**pos**. ticks	P	**perm**. sites n = 5	**temp**. sites n = 26	year of detection		
					2006	2007	2008
					n = 43	n = 83	n = 67
*Borrelia burgdorferi *s.l.	34	17.6%					
*B. valaisiana *[GU581273]	12	6.2%	2	3	x	x	x
*B. afzelii *[GU581269, GU581270]	10	5.2%	2	2	x	x	x
*B. garinii *[GU581274-GU581277]	8	4.1%	1	3	x	x	-
B. burgdorferi s.s. [GU581271, GU581272]	6	3.1%	2	3	x	x	x
Rickettsia helvetica* [JQ669952, JQ669953]	25	13.1%	4	5	x	x	x
Ca. Neoehrlichia mikurensis* [JQ669946]	20	10.5%	3	3	x	x	x
R. monacensis* [JQ669950, JQ669951]	7	3.7%	-	3	x	-	-
TBE flavivirus [JQ669945]	4	2.1%	1	-	-	x	-
*Anaplasma phagocytophilum *[JQ669947, JQ669948, JQ669949]	3	1.5%	2	1	-	x	-
*Bartonella *spp.	2	1.0%	2	-	-	x	-
*Babesia *EU1 (B. venatorum)* [JQ669954]	1	0.5%	1	-	-	-	x

***Total***	*77 *	*39.9%*	*5*	*9*			

GenBank accession numbers are also reported.

* adult tested 191

The overall pathogen infection rate was significantly higher in females than in male ticks (46.2% vs. 29.9%; p < 0.01); considering single species, this difference was significant (p < 0.05) only for *B. burgdorferi *s.l., *B. garinii *and *R. helvetica*. All pathogens were detected in the permanent sites examined, with the exception of *R. monacensis *which was only detected in temporary sites (Table 3). Whilst highly prevalent pathogens (i.e., LB agents, *R. helvetica *and "*Ca. N. mikurensis*") were detected in both permanent and temporary sites, those with low prevalence rates (e.g., TBEv, *Bartonella *spp., and *Babesia *EU1) were only detected in permanent sites (Table 3), most likely due to the high intensity of sampling. Out of 77 positive ticks, 60 (78%) harboured a single infection, 13 (17%) were co-infected by two pathogens, and 4 (5%) by three pathogens. Pathogen associations are reported in Table 4 which describes the co-infections detected in 13 female and in 4 male ticks (p < 0.05).

**Table 4:** Pathogen association in co-infected ticks

*Co-infected ticks*	Pathogen associations
double co-infection	
3	*R. helvetica-B. garinii*
3	*R. helvetica-Ca. N. mikurensis*
1	*R. monacensis-B. afzelii*
1	*R. monacensis-Ca. N. mikurensis*
1	*R. monacensis-B. valaisiana*
1	*B. afzelii-Ca. N. mikurensis*
1	*B. garinii/B. valaisiana*
1	*B. garinii-Ca. N. mikurensis*
1	TBE-*B. burgdorferi s.s*.
triple co-infection	

1	TBE-*B. burgdorferi s.s.-B. afzelii*
1	*R. monacensis-B. afzelii-Ca. N. mikurensis*
1	*R. monacensis-B. burgdorferi s.s.-Ca. N. mikurensis*

1	*B. valaisiana-Babesia *EU1-*Ca. N. mikurensis*

### Pathogen spatial and temporal distribution

Pathogen prevalence and species diversity in spatial distribution were different in the five provinces monitored (Table 5), with the Northern provinces (i.e., Udine, Pordenone, and Treviso) displaying the highest adult tick density and composition of pathogen species (Figure 1). In particular, out of 11 TTPs, 8 and 10 were detected only in Treviso and Udine, respectively. The higher overall prevalence of TTPs in Pordenone was linked specifically to infections by *B. afzelii, R. monacensis *and *"Ca. N. mikurensis"*. Despite the small number of adult ticks (Table 5) collected in the southern provinces (i.e., Verona and Vicenza), high prevalent pathogens (*R. helvetica *and *B. valaisiana*) were detected in the same areas. All pathogens except *Babesia *EU1 were detected in FVG region, whereas *R. monacensis, B. afzelii*, and TBEv were not detected in adult ticks in Veneto region.

**Table 5:** Pathogens prevalence according to province of origin (permanent and temporary sites all over the three years) and significant differences*

	*Friuli Venezia Giulia region*				*Veneto region*					
	
provinces	Pordenone n = 47		Udine n = 60		Treviso n = 64		Vicenza n = 10		Verona n = 12	
**pathogens**	**pos ticks**	**%**	**pos ticks**	**%**	**pos ticks**	**%**	**pos ticks**	**%**	**pos ticks**	**%**

Lyme agents:	14	29.8^a^	8	13.3^a^	10	15.6	-	-	2	16.7
*B. valaisiana*	3	6.4	2	3.3	5	7.8	-	-	2	16.7
*B. afzelii *	8	17.0^b^	2	3.3^b^	-	-	-	-	-	-
*B. garinii*	2	4.3	3	5.0	3	4.7	-	-	-	-
*B. burgdorferi s.s*.	2	4.3	2	3.3	2	3.1	-	-	-	-
*R. helvetica*	6	13.0	6	10.0	10	15.6	1	10.0	2	16.7
*Ca. N. mikurensis*	9	19.6	5	8.3	6	9.4	-	-	-	-
*R. monacensis*	6	13.0^c^	1	1.7^c^	-	-	-	-	-	-
TBEv	-	-	4	6.7	-	-	-	-	-	-
*A. phagocytophilum*	-	-	2	3.3	1	1.6	-	-	-	-
*Bartonella *spp	-	-	1	1.7	1	1.6	-	-	-	-
*Babesia *EU1	-	-	-	-	1	1.6	-	-	-	-

**Total**	**36**	**76.6^ABCd^**	**28**	**46.7^Ae^**	**29**	**45.3^B^**	**1**	**10.0^Ce^**	**4**	**33.3^d^**

* Equal letter corresponds to significant difference (lower case = p < 0.05; upper case = p < 0.01)

The number of pathogens identified ranged from one to seven per single site (Figure 1) and, in general, the number of ticks/site was positively correlated (R^2 ^= 0.83) with the number of pathogens detected. Interestingly, up to six pathogens were detected in 13 adults ticks collected in a single temporary site of the Treviso province. Although ticks and pathogens could be found from February to December throughout the three years of sampling, the density of adult ticks peaked in May and June, with all the 11 TTPs detected from April to June.

### Possible scenarios for tick sampling and pathogen screening

Based on the results of this study, three different tick collection scenarios were pictured, and the results obtained compared with those above. The strategies hypothesized were as follows: tick collection throughout the year and testing of female ticks only (strategy A); collection from April to June and testing of adult male and female ticks (strategy B); collection from April to June and testing of female ticks only (strategy C).

The results of the three alternative strategies are summarized in Table 6. The prevalence of TTPs assessed using these three protocols did not differ significantly from the results of the initial screening. The prevalence calculated at province level resulted in a pathogen scenario similar to that of the initial screening for strategy A and B, whereas the small number (n = 67) of ticks collected in strategy C led to very high prevalence confidence intervals (data not shown).

**Table 6:** Pathogen prevalence according to the initial screening (all adults) and different sampling strategies (A, B, C) and prevalence difference among each strategy compared to the initial screening (Δ)

			*Strategy A*			*Strategy B*			*Strategy C*		
			
Pathogens	all adults n = 193		female ticks all year n = 95		Δ	all ticks April-June n = 127		Δ	female ticks April-June n = 67		Δ
	**pos**	**%**	**pos**	**%**	***%***	**pos**	**%**	***%***	**pos**	**%**	***%***
*B. burgdorferi s.l*.	34	17.6	23	24.2	*6.6*	19	15.0	*2.6*	14	20.9	*3.3*
*B. valaisiana*	12	6.2	6	6.3	*0.1*	6	4.7	*-1.5*	4	6.0	*-0.2*
*B. afzelii*	10	5.2	7	7.4	*2.2*	4	3.1	*-2.0*	2	3.0	*-2.2*
*B. garinii*	8	4.1	7	7.4	*3.2*	7	5.5	*1.4*	6	9.0	*4.1*
*B. burgdorferi s.s*.	6	3.1	5	5.3	*2.2*	4	3.1	*0.0*	4	6.0	*2.9*
*R. helvetica*	25	13.1	18	19.1	*6.1*	20	15.7	*2.7*	14	21.2	*8.1*
*Ca. N. mikurensis*	20	10.5	8	8.5	*-2.0*	11	8.7	*-1.8*	5	7.6	*-2.0*
*R. monacensis*	7	3.7	3	3.2	*-0.5*	2	1.6	*-2.1*	1	1.5	*-2.2*
TBEv	4	2.1	4	4.2	*2.1*	4	3.1	*1.1*	4	6.0	*3.9*
*A. phagocytophilum*	3	1.6	3	3.2	*1.6*	3	2.4	*0.8*	3	4.5	*2.9*
*Bartonella *spp.	2	1.0	0	0.0	*-1.0*	2	1.6	*0.5*	0	0.0	*-1.0*
*Babesia *EU1	1	0.5	0	0.0	*-0.5*	1	0.8	*0.3*	0	0.0	*-0.5*

**Total**	**77**	**39.9**	**44**	**46.3**	***6.4***	**50**	**39.4**	***-0.5***	**31**	**46.3**	***6.4***

The occurrence of all the 11 pathogens was confirmed by strategy B, while strategies A and C did not allow detection of sporadic pathogens (i.e., *Bartonella *spp., *Babesia *EU1), which were exclusively harboured by male ticks in this study. Out of 14 sites where pathogens were detected in the initial screening, 13, 11, and 10 were positive for pathogens using strategy A, B and C, respectively. The decrease in the number of ticks screened resulted in a loss of pathogen species detected in each single site. In particular, strategies A, B, and C did not allow the detection of 1-2 pathogens in 7, 3 and 7 sites, respectively.

Estimated costs (i.e., laboratory, travel and staff expenses) for the three strategies proposed are illustrated in Table 7. Compared with the initial screening, the costs of alternative strategies A, B and C were reduced by approximately one third, half and two thirds, respectively. Pros and cons of each strategy are illustrated in Table 8.

**Table 7:** Estimated costs (€) of different tick sampling strategies and pathogen screening for a three year study

	*All ticks*		*Strategy A*		*Strategy B*		*Strategy C*	
	
	n	€	n	€	n	€	n	€
	
DNA/RNA extraction (x2)	388	3706	190	2438	254	1824	134	1286
biomolecular analyses	1018	7010	510	3516	669	4608	359	1634
sequencing	101	1818	59	1062	81	1458	55	990
draggings (travel costs)	146	24000	146	24000	71	9000	71	9000
Staff								
1 grant (sampling)	96	7234	96	7234	36	2713	36	2713
1 entomologist	32	4874	16	2399	21	3207	11	1692
1 technician	64	7932	32	3905	42	5220	22	2754
1 biotechnologist	112	26507	56	13277	75	17758	41	9697
**Total** **(reduction of costs)**		**83081**		**57832** **(30%)**		**45788** **(45%)**		**29765** **(64%)**

**Table 8:** Pros and cons of strategies A, B, and C in terms of results and costs

Strategies description	PROS	CONS
**Strategy A** (pathogen detection in female ticks collected all over the year)	Good general pathogen detection in the area Good identification of risk sites Good pathogen prevalence assessment	No detection of sporadic pathogens High loss of single pathogen detections per site Low reduction of general costs (30%) No reduction of travel costs

**Strategy B** (pathogen detection in all ticks collected in the period April-June)	Excellent pathogen detection in the area Excellent pathogen prevalence assessment Low loss of single pathogen determination per site High reduction of travel costs (62%)	Medium efficiency in identifying risk sites Low reduction of laboratory costs (33%)
	Detection of sporadic pathogens	

**Strategy C** (pathogen detection in female ticks collected in the period April-June)	Good general pathogen detection in the area High reduction of general and specific costs (64%)	Low efficiency of pathogen prevalence assessment at local level Non detection of sporadic pathogens High loss of pathogen detection per site

## Discussion

The collection of adult ticks over a three-year period combining the use of permanent and temporary sampling sites provided relevant information on the occurrence of pathogens in the area under investigation. Up to 11 pathogens were detected in about 40% of *I. ricinus *individuals sampled from north-eastern Italy, with one or more pathogens occurring in 14 collection sites. The pathogens detected in the present study had already been identified from 1989 to date in *I. ricinus *collected in the same area [3,21-32], with the exception of *B. lusitaniae*, which was detected once in nymphs [33], and *B. divergens *which was isolated from cattle only [34]. However, this study reports a comprehensive survey of TTPs occurring at one time in this area.

LB agents and *Rickettsia *species were the most prevalent pathogens in ticks and are therefore regarded as the most likely transmissible agents to animals and humans in this area. The study monitored and confirmed the occurrence of other emergent pathogens, such as *A. phagocytophilum*, and *Babesia *EU1. Interestingly, it also ascertained the presence and the distribution of "*Ca. N. mikurensis" *for the first time in Italy. The relevant prevalence of ticks positive to "*Ca. N. mikurensis" *(more than 10%) is of particular interest considering the role of this pathogen as the aetiological agent of human infections in Germany, Switzerland, and Sweden [35-37] and in a dog in Germany [19]. Indeed, following the primary isolation from rats (*Rattus norvegicus*) and *Ixodes ovatus *ticks [38] in Japan, this bacterium has been included in the list of emerging pathogens in Europe [39]. TBEv and *A. phagocytophilum *were detected in a few sites of those monitored (Table 3). The low prevalence and the scattered distribution patterns recorded for these agents, which often occur in local *foci *of transmission [40,41], complicates monitoring of tick vectors, calling for the use of other tools, such as serological methods and clinical case reports, for supporting surveillance strategies. *Bartonella *spp. was also detected in *I. ricinus *and, in spite of the increasing number of infections reported in ticks [42,43], the role played by *I. ricinus *in the transmission of this pathogen to animals and humans is disputable. However, recent laboratory evidence showed that the transmission of *Bartonella birtlesii *by *I. ricinus *ticks may occurr in naive mice [44].

Twenty-seven percent of positive ticks displayed co-infections by two or even three pathogens. Co-infections have been frequently reported in Europe not only in questing ticks [45,43,48], but also in ticks removed from humans [49], as well as domestic and wild animals [50,51].

Co-infections in questing *I. ricinus *confirm the wide host range of this tick species and the role played by mammals, such as small rodents, or birds, as reservoirs of several pathogens simultaneously. The frequent finding of co-infections in adult ticks should stimulate an increased awareness of physicians and veterinarians of potential multiple infections in vertebrate hosts, leading to different or atypical clinical presentations [52].

The present study indicates that screening of adult ticks is a successful strategy to maximize the probability of pathogen detection. The rationale for monitoring adult ticks is that the pathogen rate of infection in adult questing ticks is usually higher than in nymphs, as a consequence of the transtadial transmission of agents accumulated during the blood meal on different hosts [52].

However, despite the fact that the original screening strategy was focussed on a relatively small number of adult ticks, this strategy had considerable costs (table 7). Hence, other sampling strategies were hypothesized *a*-*posteriori*, in order to evaluate their effeciency in terms of data collected and reduction of costs. Reducing the sampling time to three months (strategies B and C) instead of the whole year, decreased costs consistently (i.e., travel and staff costs), by reducing the draggings from the initial 146 to 71. Nonetheless, strategy C resulted in a loss of data, especially at local level (provinces and sites).

Specific screening of female ticks (strategies A and C) was justified by the higher pathogen rate of infection found in *I. ricinus *females compared to males. Nevertheless, the screening of females only resulted in the fact that sporadic pathogens were not detected.

Strategy B (processing of all adult ticks from April to June) was the most cost-effective choice, and represented the best compromise for both cost reduction and reliability of results (Table 8). Therefore, this strategy is recommended as basis for circulation studies of TTPs in this specific context. However, other areas characterized by different climate, tick dynamics, and pathogen prevalence may need modifications in terms of sample size and time of tick collection.

## Conclusions

The actions that should be planned in a surveillance programme vary according to objectives (e.g., detection of major zoonotic pathogens only or emergent ones as well), ecological characteristics of pathogens to examine, estimation of costs, and budget availability. When dealing with a TBD, systematic tick collections should be undertaken in order to assess the size of the vector population and the pathogen infection rates. According to the European Center for Diseases Control [53] local, national and international health authorities should control the occurrence of a given vector-borne disease, e.g. endemic or non-endemic diseases.

This study indicates that, in the ecological landscape of north-eastern Italy, a complete picture of TTPs occurrence and of areas at risk of transmission can be drawn by systematic screening of adult ticks throughout a three-year time frame. These data can support decision makers to plan further surveillance activities. Nevertheless, tick collection and pathogen detection are expensive, especially in areas where several zoonotic TTPs coexist. Strategy B here proposed proved to fulfil the original aims of the study, being also cost-effective.

In addition, a thoughtful optimization of the diagnostic procedures could contribute to reduce costs, enabling a comprehensive, cost-effective, broad *spectrum *detection platform. Under the above circumstances, advanced biomolecular technologies, such as suspension array, reverse line blot hybridization, and novel sequencing technologies (e.g., pyrosequencing or next generation sequencing), have opened new perspectives towards maximizing results and reducing costs at the same time. The use of more sensitive approaches is likely to increase the number of pathogen species detected, as well as of co-infections diagnosed in a given area.

## Competing interests

G. Capelli and DO are members of the Bayer CVBD World Forum.

The authors declare that there is no conflict of interest regarding the present work and that the sponsor had no role in study design, data collection and analysis, decision to publish, or preparation of the manuscript.

## Authors' contributions

GC and FM conceived the study. FM, RC, and SC conducted the field study and the identification of ticks. SR, EP, AMB, SC, AS, GC, RC performed the biomolecular analyses and sequencing. GC, SR, and DO wrote the paper. All the authors read and approved the final manuscript.
